# The BHMT-betaine methylation pathway epigenetically modulates oligodendrocyte maturation

**DOI:** 10.1371/journal.pone.0250486

**Published:** 2021-05-11

**Authors:** Sarah Sternbach, Nicole West, Naveen K. Singhal, Robert Clements, Soumitra Basu, Ajai Tripathi, Ranjan Dutta, Ernest J. Freeman, Jennifer McDonough

**Affiliations:** 1 School of Biomedical Sciences, Kent State University, Kent, Ohio, United States of America; 2 Department of Chemistry and Biochemistry, Kent State University, Kent, Ohio, United States of America; 3 Department of Biological Sciences, Kent State University, Kent, Ohio, United States of America; 4 Lerner Research Institute, Cleveland Clinic, Cleveland, Ohio, United States of America; Ludwig-Maximilians-Universitat Munchen Adolf-Butenandt-Institut, GERMANY

## Abstract

Research into the epigenome is of growing importance as a loss of epigenetic control has been implicated in the development of neurodegenerative diseases. Previous studies have implicated aberrant DNA and histone methylation in multiple sclerosis (MS) disease pathogenesis. We have previously reported that the methyl donor betaine is depleted in MS and is linked to changes in histone H3 trimethylation (H3K4me3) in neurons. We have also shown that betaine increases histone methyltransferase activity by activating chromatin bound betaine homocysteine S-methyltransferase (BHMT). Here, we investigated the role of the BHMT-betaine methylation pathway in oligodendrocytes. Immunocytochemistry in the human MO3.13 cell line, primary rat oligodendrocytes, and tissue from MS postmortem brain confirmed the presence of the BHMT enzyme in the nucleus in oligodendrocytes. BHMT expression is increased 2-fold following oxidative insult, and qRT-PCR demonstrated that betaine can promote an increase in expression of oligodendrocyte maturation genes SOX10 and NKX-2.2 under oxidative conditions. Chromatin fractionation provided evidence of a direct interaction of BHMT on chromatin and co-IP analysis indicates an interaction between BHMT and DNMT3a. Our data show that both histone and DNA methyltransferase activity are increased following betaine administration. Betaine effects were shown to be dependent on BHMT expression following siRNA knockdown of BHMT. This is the first report of BHMT expression in oligodendrocytes and suggests that betaine acts through BHMT to modulate histone and DNA methyltransferase activity on chromatin. These data suggest that methyl donor availability can impact epigenetic changes and maturation in oligodendrocytes.

## Introduction

Multiple sclerosis (MS) is a progressive demyelinating disease that primarily affects the central nervous system (CNS) by means of inflammation and neurodegeneration. A hallmark of MS is the death of oligodendrocytes, the cells responsible for wrapping axons in myelin in the CNS and maintaining a healthy sheath. In demyelinating diseases like MS, oligodendrocyte progenitor cells (OPCs) fail to differentiate and make more myelin, resulting in sclerotic lesions [1,2]. It has previously been proposed that OPCs encounter a “differentiation block” or inability to effectively differentiate from OPC to myelinating oligodendrocyte in lesions of chronic MS patients [3]. Overcoming this differentiation block and promoting the differentiation of OPCs and generation of myelin is of great interest as a novel MS therapy.

**Fig 1:**
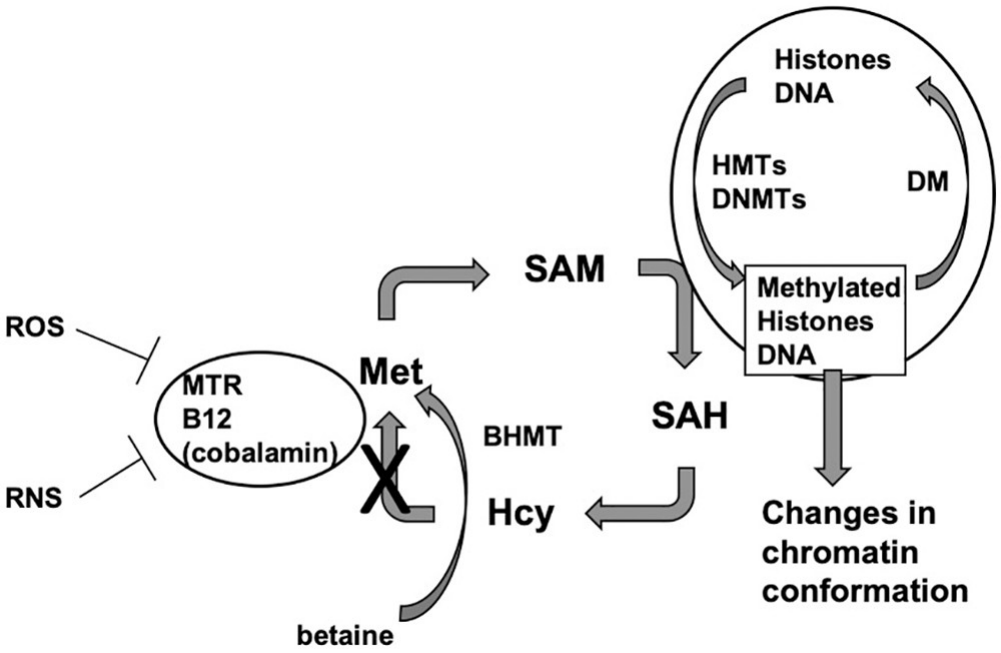
Methionine metabolism. In the methionine cycle, homocysteine is remethylated to methionine through the methionine synthase enzyme (MTR) with vitamin B_12_ as a cofactor, or necessary catalyst for MTR activity. Methionine is then converted to SAM, which donates a methyl group and is converted to S-adenosylhomocysteine (SAH), which is then converted back to homocysteine. Histone and DNA methyltransferases transfer the methyl group from SAM to amino acids and bases on histone amino-terminal tails and to the 5 position of cytosine residues in DNA [11]. When MTR enzymatic activity is inhibited by reactive oxygen and nitrogen species, BHMT uses betaine to remethylate homocysteine to methionine. Betaine can be taken in through the diet or synthesized through the oxidation of choline in mitochondria. Abbreviations: Hcy, homocysteine; Met, methionine; SAM, S-adenosylmethionine; SAH, S-adenosylhomocysteine; BHMT, betaine homocysteine methyltransferase; ROS, reactive oxygen species; RNS, reactive nitrogen species; HMTs, histone methyltransferases; DNMTs, DNA methyltransferases.

The etiology and pathogenesis of MS are not well understood, though research suggests that there is interplay between different immunological, environmental, and genetic factors [4]. We have previously shown there is a dysregulation of methionine metabolism in both the CNS and periphery in MS [5,6]. Specifically, increased reactive nitrogen species (RNS) inhibit methionine synthase (MTR) enzymatic activity [7], causing levels of betaine and S-adenosylmethionine (SAM) to decreased in MS cortical tissue, leading to subsequent changes in methylation and gene transcription. Betaine-homocysteine S-methyltransferase (BHMT) is an enzyme in the methionine cycle that was previously thought to be restricted to the cytoplasm of hepatocytes and kidney cells but has recently been implicated in MS pathology in the brain [5,8–10]. BHMT is an enzyme that catalyzes the remethylation of homocysteine (Hcy) to methionine with betaine as the methyl donor. Methionine is then converted to SAM, the key methyl donor required for histone and DNA methylation (Fig 1). When MTR activity is downregulated, levels of SAM are also decreased. However, BHMT, using betaine as a substrate, bypasses the downregulation of MTR and restores SAM levels [5]. This BHMT-betaine methylation pathway ensures the availability of SAM for a variety of DNA and histone methylation processes.

OPC survival and differentiation are dependent upon DNA and histone methylation, and both processes require SAM [12–16]. It is widely known and accepted that DNA methyltransferases (DNMTs) and histone methyltransferases (HMTs) usually act as gene silencers, methylating specific residues on histones and DNA. The process of OPC differentiation is tightly regulated through concerted action of gene repression and activation, i.e., repression of multipotency genes and activation of myelin genes [15]. In addition, research has shown that histone acetylation favors a shift in OPCs back to a multipotent state [16]. This idea of necessary regulated periods of repression and activation is consistent with both developmental myelination and later remyelination processes [13,15,17–19]. DNMTs can be categorized into two distinct methylation enzymes: DNMT1, which is important during development for maintaining methylation patterns, and DNMT3A/B, an important regulator of *de novo* or “new” methylation [14–16]. *De novo* methylation through DNMT3A/B is responsible for establishing new methylation patterns, specifically for the differentiation of OPCs into myelinating oligodendrocytes following injury or stress [16,18]. HMTs can also be classified into two categories: lysine specific and arginine specific. Lysine specific HMTs transfer 1–3 methyl groups from SAM to histone tails to be mono-, di- or tri- methylated, while arginine specific HMTs transfer 1–2 methyl groups for mono-methylation or symmetric/asymmetric di-methylation [20]. Histone methylation is a dynamic process, and concerted interactions between HMTs and histone demethylases control gene expression [21]. How DNMT and HMT activities can be regulated or controlled following oxidative insult remains unknown.

MS is the leading neurological disorder among young adults affecting over two million people, globally [22]. Recent research has turned to epigenetics for neurological disease protection and prevention [23–26]. The present study was undertaken to determine the presence of the BHMT enzyme in oligodendrocytes and the role of the BHMT–betaine pathway on chromatin regulation and HMT/DNMT activity in OPCs. This is the first report of BHMT in oligodendrocytes and the BHMT-betaine methylation pathway in epigenetic modulation of OPC gene transcription.

## Materials and methods

Research with human MS tissue was approved by the Cleveland Clinic/Lerner Research Institute IRB with written consent. The data in this paper were analyzed anonymously.

### Cell culture

The MO3.13 human glial (oligodendrocytic) cell line and primary oligodendrocyte cultures were used to study BHMT and the BHMT-betaine pathway in oligodendrocytes. MO3.13 oligodendrocyte cells (Cellutions Biosystems Inc., CLU301) cells were cultured in DMEM supplemented with 10% FBS and 1% Penicillin/Streptomycin and 100 nM phorbol 12-myristate 13-acetate (PMA) was used for 6 days to promote oligodendrocyte maturation. Rat primary oligodendrocytes were cultured using previously described methods [27]. MS-like conditions were induced using sodium nitroprusside dihydrate (SNP) (Millipore Sigma, Burlington, MA, 71778), a nitric oxide (NO) donor, at 400 μM overnight in MO3.13 cells. Betaine treatment was overnight at a concentration of 1 mM [4] (Millipore Sigma, Burlington, MA, 61962).

### Immunohistochemistry

Cover slips with MO3.13 and primary rat oligodendrocytes were fixed with 4% PFA and blocked in 3% normal donkey serum. Cover slips were then incubated overnight in either BHMT (1:500, Santa Cruz Biotechnology, sc69708) and OLIG2 (1:250, Abcam, Cambridge, MA, ab136253), or BHMT and MBP (1:200, Santa Cruz Biotechnology, sc271524) at 4°C and incubated in the appropriate secondary antibody the following day for 3 hours at 4°C. After incubation with secondary antibodies, cover slips were washed with PBS and mounted on microscope slides using Vectashield Antifade Mounting Medium with 4’, 6-diamidino-2-phenylindole (DAPI) (Vector Laboratories, Burlingame, CA, H-1200). Images were acquired with an Olympus FV1000 confocal microscope. Image stacks were z-projected with ImageJ (National Institutes of Health) and channels merged to show colocalized signals. A slide of MS postmortem brain tissue was obtained from the brain bank at the Cleveland Clinic. The section was deparaffinized with xylenes, rehydrated, and antigen retrieval was performed by placing the section in 10 mM sodium citrate buffer in an 800 W microwave oven for 2 minutes. The section was blocked in 5% normal donkey serum in TBS with 0.025%Triton-X-100 (TBS-T) for 2 hours and incubated overnight at 4°C in an antibody to BHMT (1:200) in TBS-T + 5% donkey serum followed by biotinylated anti-mouse secondary antibody (1:500) in TBS-T + 5% donkey serum. Avidin biotin complex (Vector Laboratories, PK-6100) in PBS was added for 30 min, followed by a 10 min incubation in 3,3′-Diaminobenzidine (DAB) chromogen (Vector Laboratories, SK4100). The section was thoroughly rinsed in PBS and blocked in 5% donkey serum for 1 hour to be prepared for sequential immunohistochemistry staining with a second antibody. For the sequential stain, the section was incubated in Olig2 antibody (1:500) in TBS-T + 5% donkey serum overnight at 4°C. On the third day, the section was washed with PBS, incubated for 1 hour in secondary antibody (1:500) at room temperature, followed by a 30 min incubation with avidin biotin complex (Vector Laboratories, AK-5200) in PBS and 30 min incubation in Alkaline Phosphate (AP) Blue (Vector Laboratories, SK-5300) chromogen. The section was thoroughly washed in PBS and coverslipped with permount mounting medium. Images were acquired on a brightfield microscope. For proteolipid protein (PLP) immunohistochemistry, human MS brain paraffin sections were deparaffinized and rehydrated followed by antigen retrieval (10mM citrate buffer, pH-6). After washing in PBS, peroxidase enzyme was deactivated using 3% H_2_O_2_ in PBS-T. Section was blocked in 5% normal goat serum in PBS-T for an hour at room temperature, followed by overnight incubation in rat anti-PLP (1:250) at room temperature. After PBS washing, sections were incubated in biotin tagged secondary antibody (1:500) for 1 hour followed by Avidin-Biotin complex staining as per manufacturer’s suggestion (Vector Laboratories, PK-6100). Sections were washed in PBS, developed with DAB (Sigma, Burlington, MA, D5905) and 0.012% H_2_O_2_, dehydrated and mounted before imaging on a bright field microscope.

### Western blotting

To determine whether BHMT interacts with chromatin in oligodendrocytes, we performed chromatin fractionation from MO3.13 cells. Control cells and cells treated overnight with 400 μM SNP were scraped at 100% confluency, and nuclear fractions were made and separated using buffers with increasing salt concentrations (0.3 M– 1.8 M NaCl) according to O’Hagan et al., 2012 [28]. In these experiments, proteins that are present in high salt fractions (1.2 M-1.8 M NaCl) are more tightly interacting with chromatin [28]. Each fraction was collected and Western blotted with antibodies to BHMT (1:500, sc-69708, Santa Cruz Biotechnology), DNMT3a (1:1000, Cell Signaling Technology, Danvers, MA, 3598S), and H3K4me3 (1:500, Abcam, Cambridge, MA, ab8580). Additional MO3.13 cells were treated with 400 μM SNP overnight and whole cell lysate was made using 1X RIPA buffer (Sigma, 20–188). Proteins were run on an SDS gel and Western blotted with the BHMT antibody. Densitometry was performed with ImageJ. Relative protein levels were normalized to GAPDH (1:1000, Millipore Sigma, Burlington, MA, MAB374). Results are from three separate experiments and statistical significance was determined by a two-tailed Student’s t-test, p ≤ 0.05 was considered significant.

### qRT-PCR

Relative levels of mRNA for genes relating to oligodendrocyte maturation, including SRY-Box Transcription Factor 10 (SOX10), Myelin Regulatory Factor (MYRF), NK2 Homeobox 2 (NKX-2.2), Hes Family BHLH Transcription Factor 5 (HES5), and BHMT were quantitated by qRT-PCR in mRNA isolated from human MO3.13 oligodendrocytes treated with 400 μM SNP, 1 mM betaine, or 400 μM SNP and 1 mM betaine. qRT-PCR was performed with SYBR green (Agilent Technologies, Santa Clara, CA, 600886) and gene specific primers that included an intronic region. Primer sequences are included in S1 Table. Relative levels of mRNA expression in control and treated samples were determined by the 2^-ΔΔCt^ method following normalization to β-actin. Results are from nine separate experiments and statistical significance was determined by a two-tailed Student’s *t* test and the non-parametric Mann Whitney test, with p ≤ 0.05 considered significant.

### HMT and DNMT activity assay

HMT activity was quantified with an EpiQuik Histone Methyltransferase Activity/Inhibition Assay Kit (Epigentek Inc., Farmingdale, NY, P-3002-1) and total DNMT activity was quantified with an EpiQuick DNA Methyltransferase Activity/Inhibition Assay Kit (Epigentek Inc., Farmingdale, NY, P-3001-1). Briefly, MO3.13 cells were treated with either a scrambled or targeting siRNA oligonucleotide for BHMT using TransIT-X2 transfection reagent (Mirus Bio, Madison, WI, MIR6004) to show that betaine related increases in DNMT and HMT activity are BHMT-dependent (siRNA BHMT targeting sequence UUAGAACGCUUAAAUGCUG, non-targeting control sequence AUGCGACUAAACACAUCAA) (Dharmacon Inc., Lafayette, CO). Transfection lasted for 48 hours with 1 mM betaine treatment for the final 24 hours. SiRNA knockdown efficiency was ~90%, shown in S1 Fig. Cells were scraped and nuclear extracts were prepared as previously described [29]. Six μg of protein from three separate experiments were plated per well and used for the assay. Results are from six separate experiments and significance was determined by a two-tailed Student’s *t* test, with p ≤ 0.05 considered significant.

### Co-Immunoprecipitation

Protein-protein interacts were determined using a Pierce Crosslink Magnetic IP/Co-IP Kit (Thermo Fisher Scientific, Waltham, MA, 88805). MO3.13 oligodendrocytes were grown to confluency and lysed to be used for the assay (1000 μL of IP Lysis/Wash Buffer: 25 mM Tris, 150 mM NaCl, 1 mM EDTA, 1% NP40, 5% glycerol). Briefly, 10 μg of BHMT antibody was bound to Pierce Protein A/G Magnetic Beads (Santa Cruz Biotechnology, sc-69708) and crosslinked with disuccinimidyl suberate (DSS) for 30 minutes. Normal rabbit serum was used as a negative control. Non-crosslinked antibody was removed, and the beads were washed with IP Lysis/Wash Buffer before adding 500μL of cell lysate solution. The lysate was incubated on the beads at room temperature for 1 hour and each sample was sequentially eluted with IP Lysis/Wash buffer and Elution Buffer. Flow-through (FT) and immunoprecipitated (IP) samples were run on a Western blot to identify DNMT3a interactions with BHMT.

## Results

Changes in levels of methionine cycle metabolites has been shown to affect DNA and histone methylation processes and result in subsequent neurological damage [30–34]. We have previously demonstrated that the presence of the BHMT-betaine pathway in neurons bypasses the downregulation of MTR activity and effectively keeps the methionine cycle moving [35], and hypothesize a similar mechanism is occurring in oligodendrocytes.

**Fig 2:**
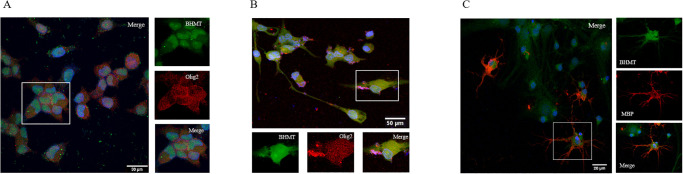
BHMT is present in oligodendrocytes. (A) Representative confocal images acquired at 60x showing immunofluorescent staining for BHMT (green) and OLIG2 (red) in immature MO3.13 oligodendrocytes. Nuclei are stained with DAPI (blue). Area outlined in white was enlarged and split channels are shown (right). BHMT immunoreactivity is co-localized in oligodendrocytes also stained with OLIG2. (B) Representative confocal images acquired at 60x showing immunofluorescent staining for BHMT (green) and OLIG2 (red) in mature MO3.13 oligodendrocytes. Nuclei are stained with DAPI (blue). Area outlined in white was enlarged and is shown (right). (C) Representative confocal images acquired at 40X showing immunofluorescent staining for BHMT (green) and MBP (red) in mature primary rat oligodendrocytes. Nuclei are stained with DAPI (blue). Area outlined in white was enlarged and shown to the right.

**Fig 3:**
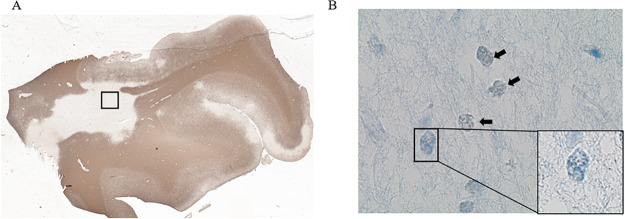
BHMT is present in a human MS lesion. (A) Postmortem MS tissue stained for PLP. A white matter lesion is evident. Area shown in (B) is from boxed white matter lesion area on adjacent section. Brightfield images taken at 40x show colocalization of OLIG2 (blue alkaline phosphatase staining) and BHMT immunoreactivity (brown DAB precipitate) denoted by arrows on cortical tissue sections. Area outlined in black was enlarged.

Staining for BHMT and the oligodendrocyte lineage marker, OLIG2, identified endogenous expression of BHMT in both the cytoplasm and nucleus of immature as well as mature MO3.13 oligodendrocytes (Fig 2A and 2B). Staining for BHMT and MBP in mature primary rat oligodendrocytes confirms the presence of BHMT in the nucleus and cytoplasm as well (Fig 2C). Researchers have reported that there is an increased recruitment of OPCs and immature oligodendrocytes to lesioned areas in the brain, but a marked inability to differentiate [3,36–38]. To determine if BHMT could play a role in oligodendrocyte differentiation in lesions, we examined BHMT expression in postmortem MS tissue. MS postmortem brain tissue was examined for BHMT expression in lesioned areas to illustrate the potential for the BHMT-betaine pathway to function in immature oligodendrocytes and promote their differentiation. The human tissue analyzed contained a large white matter lesion which was visualized by immunohistochemistry for PLP (Fig 3A). Immunohistochemistry on adjacent sections to BHMT and OLIG2 shows immunopositive cells within the lesioned area (Fig 3B). By definition, lesions lack myelinating oligodendrocytes; therefore, these cells may be OPCs or immature oligodendrocytes that have migrated to the lesion and do not have the capacity to differentiate with insufficient levels of betaine.

Future studies with additional human tissue samples will be necessary to confirm these findings.

**Fig 4:**
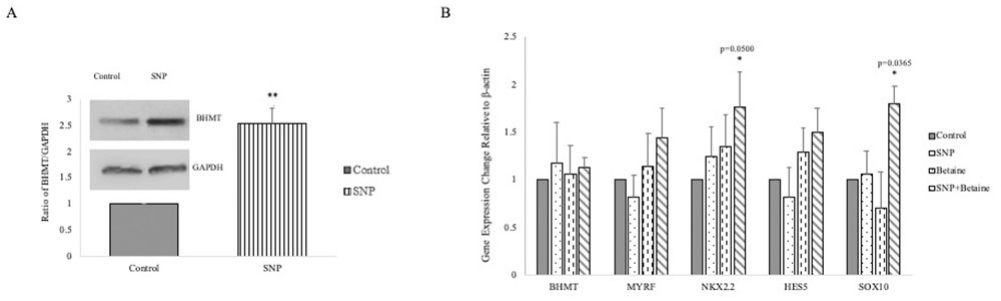
The BHMT-betaine pathway is involved in regulating differentiation in oligodendrocytes. (A) Representative Western blot performed with control and SNP treated MO3.13 oligodendrocytes. Lysates were probed for BHMT and the cytoplasmic marker GAPDH. Error bars are expressed as SEM. **, p = 0.007. (B) Changes in gene expression were confirmed by qRT-PCR for select transcripts that play a role in regulation of OPC differentiation. qRT-PCR was performed with RNA isolated from control MO3.13 oligodendrocytes and oligodendrocytes treated with 400 μM SNP, 1 mM betaine, or SNP/betaine. Relative expression levels with controls set at 1 are shown. Error bars indicate SEM, n = 9. P values shown were determined by a two-tailed Students t-test. Additionally, a Mann-Whitney U nonparametric test shows that SOX10 expression treated with SNP and betaine is increased, p = 0.016.

MS pathology presents as an increase in oxidative stress and increased RNS resulting from activated microglia in the CNS. This increase in RNS is accompanied by a decrease in MTR activity [7], as shown in Fig 1, and the BHMT-betaine pathway may exert a compensatory effect over the entire methionine cycle. Therefore, we investigated whether oxidative damage could increase BHMT expression. Western blotting shows that levels of BHMT increase by two-fold in MO3.13 oligodendrocytes treated with the NO donor SNP (Fig 4A). However, qRT-PCR analysis revealed that BHMT mRNA expression was unchanged, suggesting that betaine regulates BHMT post-transcriptionally. qRT-PCR analysis of key regulators that drive OPC differentiation showed that simultaneous treatment with SNP and betaine increased the relative expression of genes that promote maturation of OPCs (*SOX10*, p = 0.036 and *NKX-2*.*2*, p = 0.05), but had no effect on *HES5* which inhibits OPC differentiation [39] (Fig 4B). There were no changes in expression of these genes when cells were treated with only SNP or betaine alone, indicating that betaine treatment increases gene expression under conditions of oxidative stress.

**Fig 5:**
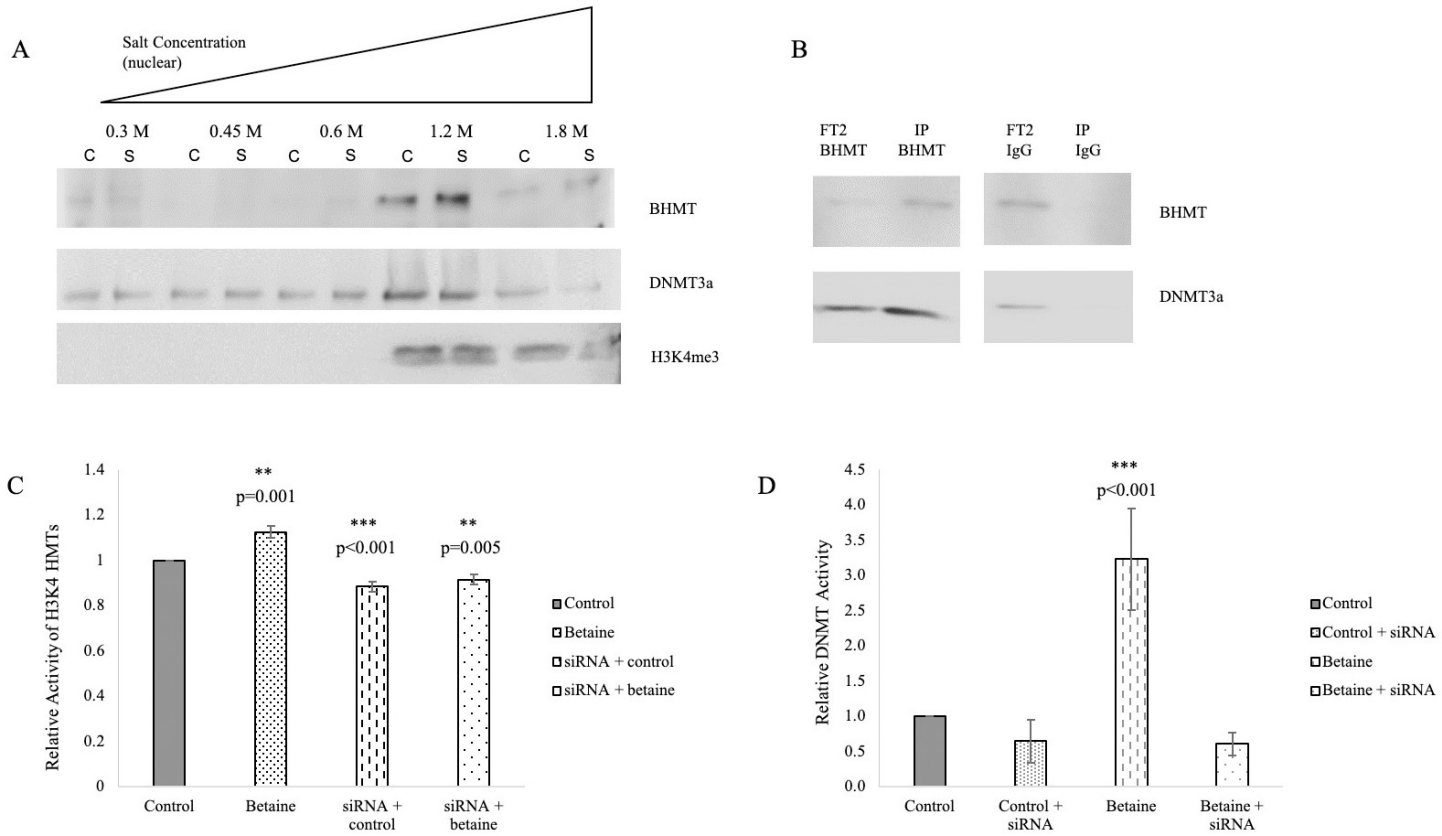
The BHMT–betaine pathway regulates HMT and DNMT activity. (A) Representative Western blot images for BHMT, DNMT3a, and H3K4me3 from nuclear chromatin fractions of MO3.13 oligodendrocytes treated with 400 μM SNP. Lanes are denoted “C” for control and “S” for SNP treated. n = 3. (B) Co-IP analysis of protein-protein interactions between BHMT and DNMT3a in MO3.13 oligodendrocytes. Co-IPs were performed using an antibody to BHMT. Flow-through (FT) and IP samples were run on a Western blot to identify DNMT3a interactions with BHMT. Normal rabbit serum (IgG) was used as a negative control. n = 2. (C) Quantitation of HMT activity on H3K4 in control MO3.13 oligodendrocytes and oligodendrocytes transfected with siRNA to BHMT, with and without 1 mM betaine. Error bars indicate SEM, n = 6. (D) Quantitation of total DNMT activity in control MO3.13 oligodendrocytes and oligodendrocytes transfected with siRNA to BHMT, with and without 1 mM betaine. Error bars indicate SEM, n = 6.

To examine more closely the role of BHMT in the nucleus, we performed chromatin fractionation followed by Western blotting to look at the localization of various proteins on chromatin with and without SNP-induced oxidative stress (Fig 5A). In these experiments, proteins present in higher salt fractions are more tightly bound to chromatin. We found that BHMT, DNMT3a, and H3K4me3 are all bound to tighter chromatin fractions (1.2–1.8 M NaCl), indicating that BHMT has the potential to interact with these proteins on chromatin. To further investigate this relationship, we performed co-IP studies and found that BHMT interacts with DNMT3a in immature oligodendrocytes (Fig 5B). BHMT interacting with this DNMT is significant as it provides a mechanism of action for the BHMT-betaine pathway in regulation of gene expression. Specifically, the interaction between BHMT and DNMT3a is important because DNMT3a is implicated in *de novo* methylation, which is required for remyelination.

Research has shown that increased levels of NO downregulates myelin gene expression [40]. In addition, we have found that SNP treatment decreases HMT activity in immature oligodendrocytes and that treatment with SNP and betaine restores HMT activity (S2 Fig). Since DNA and histone methylation play a key role in regulation of gene expression, we investigated the specific effects of betaine and the BHMT-betaine pathway on DNMT and HMT activity in immature oligodendrocytes. We found that betaine significantly increased HMT activity on H3K4; however, knockdown of BHMT expression with an siRNA blocked the regulation of HMT activity by betaine (Fig 5C). We also quantitated total DNMT activity with and without betaine, and again found that the knockdown of BHMT failed to produce the same increase in activity (Fig 5D). The siRNA mediated knockdown of BHMT shows that betaine mediated regulation of DNMT and HMT activity is BHMT-dependent. These localization and interaction studies provide evidence of regulation of HMT and DNMT activity through the BHMT-betaine pathway.

## Discussion

Here we demonstrate for the first time that the BHMT-betaine pathway can regulate DNMT and HMT activities and expression of genes involved in oligodendrocyte maturation under conditions of oxidative stress. Based upon in vitro immunofluorescent and immunohistochemical staining, we concluded that BHMT is present in oligodendrocytes in both rats and humans, specifically in human MS tissue. This is a novel finding as BHMT has not yet been shown in oligodendrocytes. Our data suggest that activation of the BHMT-betaine methylation pathway by administering betaine may overcome the differentiation block of immature OPCs that occurs under oxidative conditions. Betaine increased the expression of transcription factors necessary for oligodendrocyte differentiation (*SOX10*, *NKX-2*.*2*) under conditions of oxidative stress. Mechanistically, our data suggest that the regulation of these genes by betaine could be a result of changes in DNA and histone methylation which are known to be required for oligodendrocyte development [12,41]. The increase in BHMT under oxidative conditions, coupled with the supplementation of betaine, allows an increase in the methylation potential or SAM/SAH ratio, which would activate HMTs and DNMTs.

A role for the BHMT-betaine pathway in regulating methylation of DNA is supported by our chromatin fractionation and co-IP studies that demonstrate an interaction between BHMT and DNMT3a on chromatin. Consistent with these findings, we also showed that the BHMT-betaine pathway is responsible for modulating total DNMT activity and HMT activity on H3K4 residues, and that betaine administration increases these activities in MO3.13 oligodendrocytes. These data suggest that betaine may silence transcription for some subsets of genes by increasing DNA methylation, while activating expression of other genes by increasing the transcriptional activator H3K4me3. The knockdown of BHMT in these assays is important because it is indicative of a specific effect that betaine has on the methionine cycle as opposed to a widespread effect of betaine administration, and demonstrates that betaine mediated effects on methylation and gene expression are dependent on BHMT. The total DNMT activity assay demonstrates a greater magnitude of change in enzymatic activity in the absence of BHMT, likely indicating that BHMT is bound to DNMTs at more sites than HMTs. This is consistent with our previous study showing that in neurons, the BHMT-betaine pathway activates the Set/MLL HMT that methylates H3K4 to H3K4me3 to regulate gene expression where BHMT is bound to chromatin [35].

Because the BHMT-betaine pathway regulates DNMTs and the Set/MLL HMT that methylates the transcriptional activator H3K4me3, it may contribute to the repression of some genes and activation of others. This regulation of H3K4me3 could explain the increase in SOX10 and NKX-2.2 expression with betaine under oxidative conditions. Another possibility is that the BHMT-betaine pathway doesn’t directly regulate the SOX10 and NKX-2.2 genes. Instead, it could be that the betaine is silencing repressors of these genes. This is consistent with the wave of repressive epigenetic regulation including increased 5mC, increased H3K9me3, and decreased histone acetylation that occurs during oligodendrocyte development. It isn’t known if NKX-2.2 is epigenetically regulated, however, it has been shown that SOX10 regulates NKX-2.2 [42]. It may be that SOX10 is driving the response to betaine and effects on NKX-2.2 are a result of increased SOX10 expression. Taken together, we believe that the BHMT-betaine pathway, as part of the methionine cycle, is responsible for epigenetic modulation of gene expression of oligodendrocyte differentiation factors, specifically through DNMTs and HMTs.

Betaine is found widely in nature and already exists as an FDA approved medication to treat homocystinuria. Increased levels of betaine and choline have been shown to have positive effects on overall CNS health, including decreases in proinflammatory cytokines and homocysteine [43]. We have previously shown that betaine can modulate gene expression in neurons and that depletion of betaine in the MS brain is linked to mitochondrial dysfunction [5]. In the EAE mouse model of MS, Yang et al. found that betaine administration lead to improved clinical scores and lessens disease severity [44]. This is consistent with our previous findings that betaine is neuroprotective in the cuprizone mouse model of MS [34]. In addition, Amiraslani et al. have shown that betaine decreases the release of NO from microglia in the brain following treatment with lipopolysaccharide through hypermethylation of the nuclear factor-kappa B (NF- κB) promoter [45]. These data are consistent with the Ili et al. study showing that betaine attenuates the effects of oxidative stress in Aβ-injected mice [46]. Taken together, these studies support our finding of betaine epigenetically modulating gene expression through the BHMT-betaine pathway.

While studies have shown that DNMT activity is important for OPC maturation and remyelination, little is known concerning the specific regulatory regions that are methylated. Moyon et al. showed an increase in overall 5-methylcytosine (5mC) in adult OPCs following lysolecithin injection, indicating an increase in DNA methylation [17]. Again, our data suggest that this increase in 5mC is occurring at specific loci where BHMT is bound, and that betaine is donating a methyl group locally to BHMT and ultimately DNMT and HMT enzymes to regulate genes involved in myelination and differentiation at that locus. This is especially important as we consider the waves of gene regulation (repression and activation) that need to occur to promote myelination. Our discovery of the BHMT-betaine methylation pathway in oligodendrocytes provides insight into how epigenetics, specifically methylation through SAM, can alter DNMT and HMT activity and specifically tailor expression of both positive and negative developmental and remyelination regulators [47–49].

Although not investigated here, ChIP-seq analysis of oligodendrocytes with BHMT will be informative as to which specific genes are influenced by the BHMT-betaine pathway. Another technical issue to consider is that the MO3.13 oligodendrocyte cell line, while human, is not a primary oligodendrocyte culture and we cannot rule out potential effects from the tumorigenic properties of the cells on enzymatic activity. Nonetheless, the MO3.13 oligodendrocytes do make endogenous BHMT and our data provides the framework to study epigenetic changes from betaine treatment. We understand that more basic research is required to determine the dosage and timing of betaine administration under oxidative conditions. However, based on the data presented here, betaine intervention provides a plausible therapy for neurodegenerative disorders.

In conclusion, we demonstrated that oligodendrocyte gene expression can be modulated by betaine supplementation through the BHMT-betaine methylation pathway, specifically by activation of DNMT and HMT enzymes. Our study suggests that dietary betaine supplementation may prove to be a therapeutic agent for MS and other demyelinating disorders.

## Supporting information

S1 Fig SiRNA knockdown efficiency in MO3.13 cells. Representative Western blot performed with control and 1 mM betaine treated MO3.13 oligodendrocytes with and without siRNA knockdown. Lysates were probed for BHMT and the nuclear marker histone H3. Band density was quantified in ImageJ, results shown to the right. Overall knockdown efficiency is ~90%.
(TIF)

S2 Fig HMT activity in MO3.13 cells. HMT activity assay on H3K4 with control, 400 μM SNP, and 400 μM + 1 mM betaine treated MO3.13 oligodendrocytes. Significance determined by a Students *t* test. Relative activity levels of the control set at 1 are shown. Error bars indicate SEM, n = 3.
(JPG)

S1 Table List of oligonucleotides used as PCR primers. (PDF)

S1 Raw images (PDF)

S1 Data (XLSX)
