# Supplementary figures and images for: The BHMT-betaine methylation pathway epigenetically modulates oligodendrocyte maturation
Source: PLoS One. 2021 May 11;16(5):e0250486. doi: 10.1371/journal.pone.0250486 (PMC8112889; doi:10.1371/journal.pone.0250486)

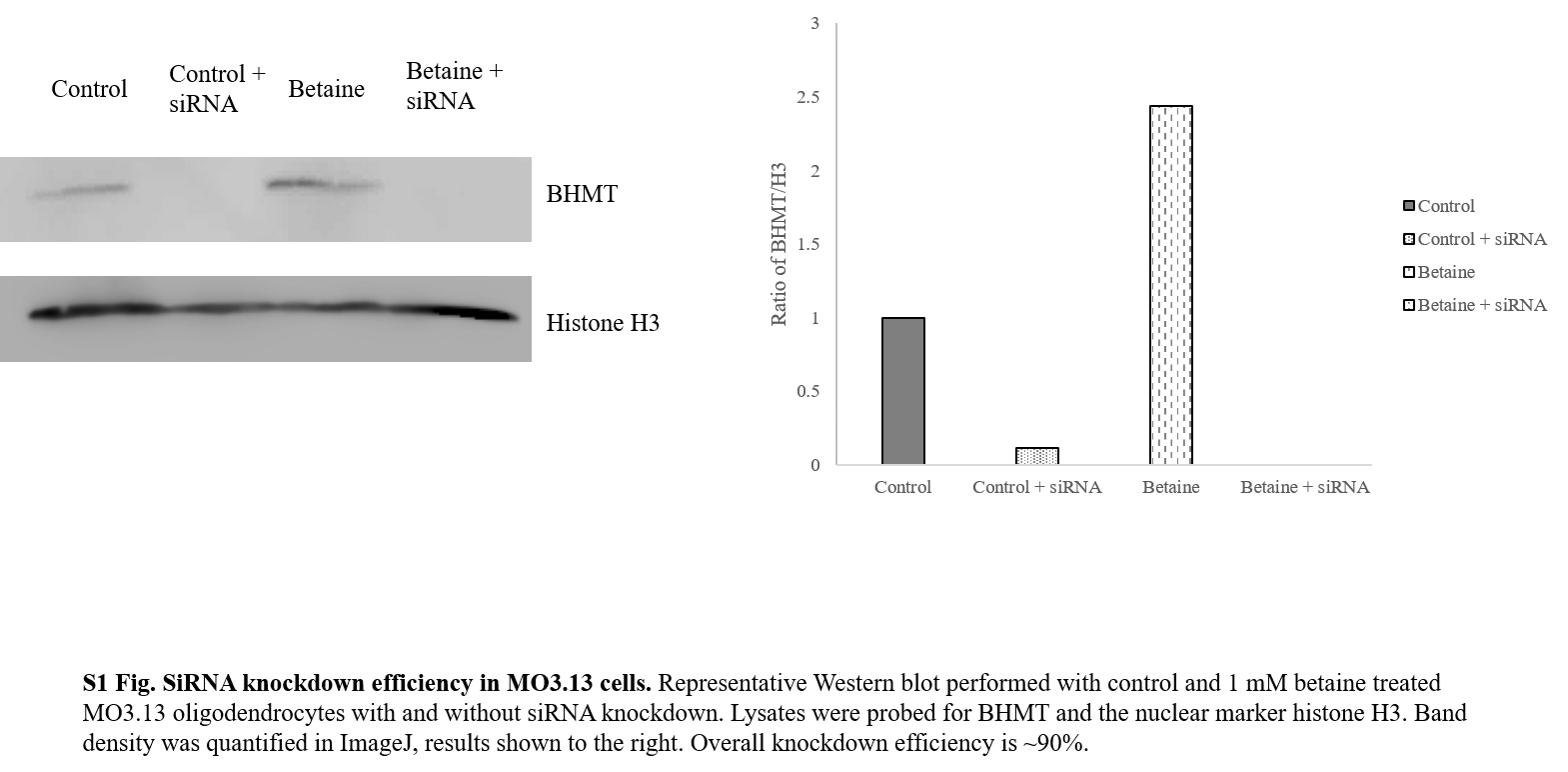

Supplement: S1 Fig — Representative Western blot performed with control and 1 mM betaine treated MO3.13 oligodendrocytes with and without siRNA knockdown. Lysates were probed for BHMT and the nuclear marker histone H3. Band density was quantified in ImageJ, results shown to the right. Overall knockdown efficiency is ~90%. (TIF) [file pone.0250486.s001.tif]

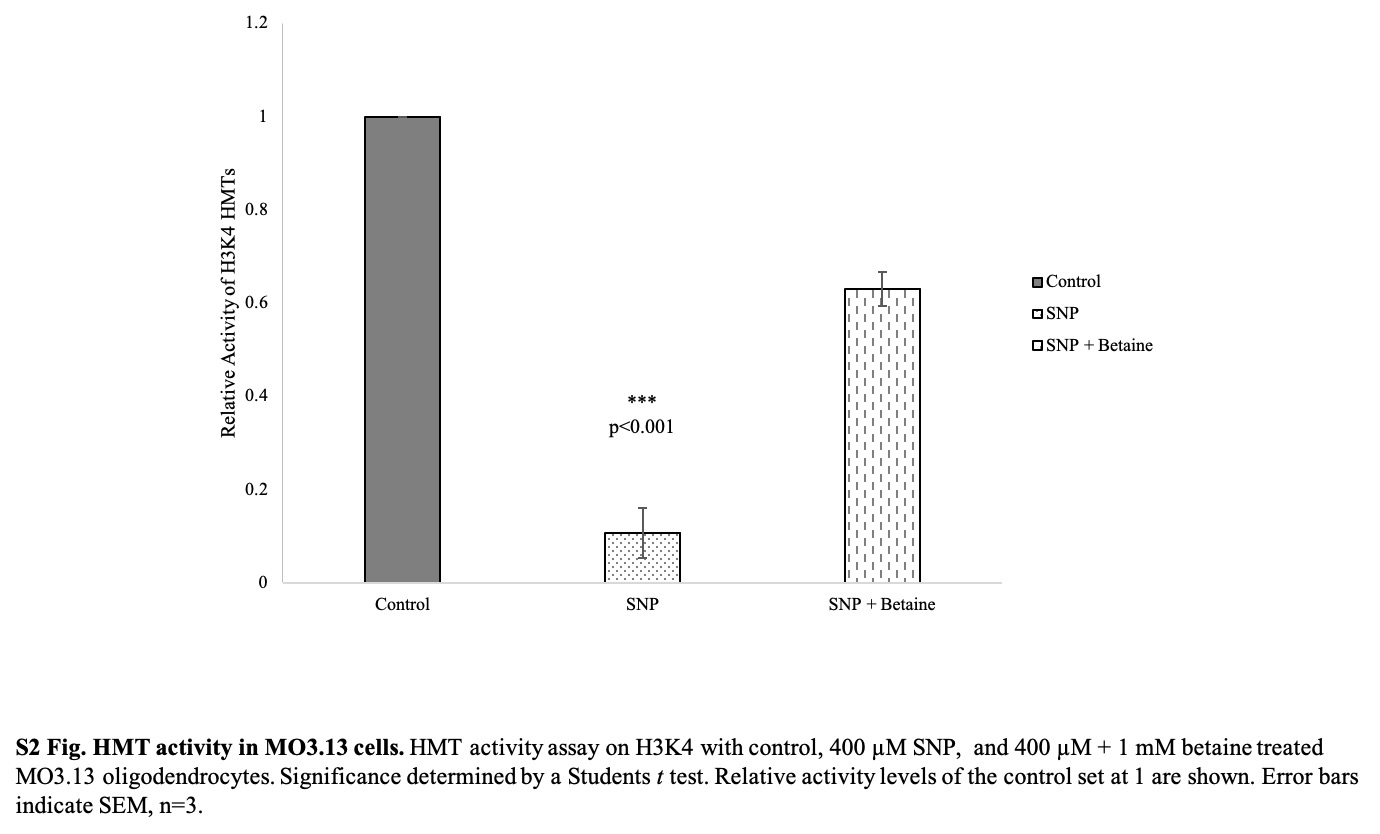

Supplement: S2 Fig — HMT activity assay on H3K4 with control, 400 μM SNP, and 400 μM + 1 mM betaine treated MO3.13 oligodendrocytes. Significance determined by a Students t test. Relative activity levels of the control set at 1 are shown. Error bars indicate SEM, n = 3. (JPG) [file pone.0250486.s002.jpg]
