# Supplementary material for: The BHMT-betaine methylation pathway epigenetically modulates oligodendrocyte maturation
Source: PLoS One. 2021 May 11;16(5):e0250486. doi: 10.1371/journal.pone.0250486 (PMC8112889; doi:10.1371/journal.pone.0250486)
Supplement: S1 Table — (PDF) [file pone.0250486.s003.pdf]

Supplemental Table 1. List of oligonucleotides used as PCR primers.

**Primer**	**Sequence**
BHMT F	5’-AGCAGGAGGAGTGAGTCAGA-3'
BHMT R	5'-CTGCCACAGGTTTACCGGAT-3'
HES5 F	5'-GCTCAGCCCCAAAGAGAAAA-3'
HES5 R	5'-GTAGTCCTGGTGCAGGCTCT-3'
MYRF F	5'-CAGTCAGCCTCTCTCCTTGC-3'
MYRF R	5'-GGGGGAGAGGAGTTCATCT-3'
NKX2.2 F	5'-AAGGTCCGGAGGAAGAGAACGA-3'
NKX2.2 R	5'-TCCGGTGACTCGTCGGC-3'
SOX10 F	5'-ATCCAGGCCCACTACAAGAGC-3'
SOX10 R	5'-ATGTCCACGTTGCCGAAGT-3'
