# Supplementary material for: The BHMT-betaine methylation pathway epigenetically modulates oligodendrocyte maturation
Source: PLoS One. 2021 May 11;16(5):e0250486. doi: 10.1371/journal.pone.0250486 (PMC8112889; doi:10.1371/journal.pone.0250486)
Supplement: S1 Raw images — (PDF) [file pone.0250486.s004.pdf]

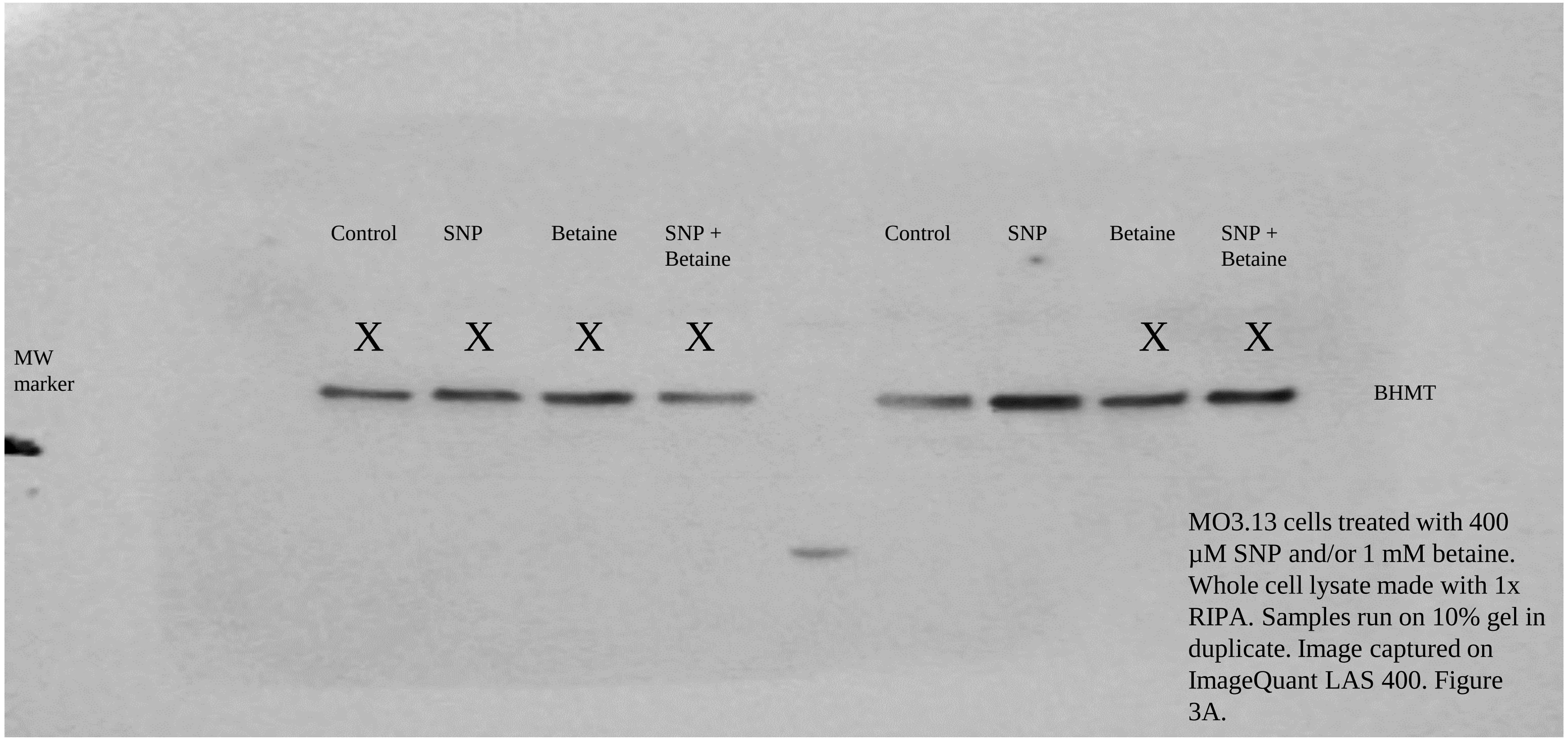

MO3.13 cells treated with 400 μM SNP and/or 1 mM betaine. Whole cell lysate made with 1x RIPA. Samples run on 10% gel in duplicate. Image captured on ImageQuant LAS 400. Figure 3A.

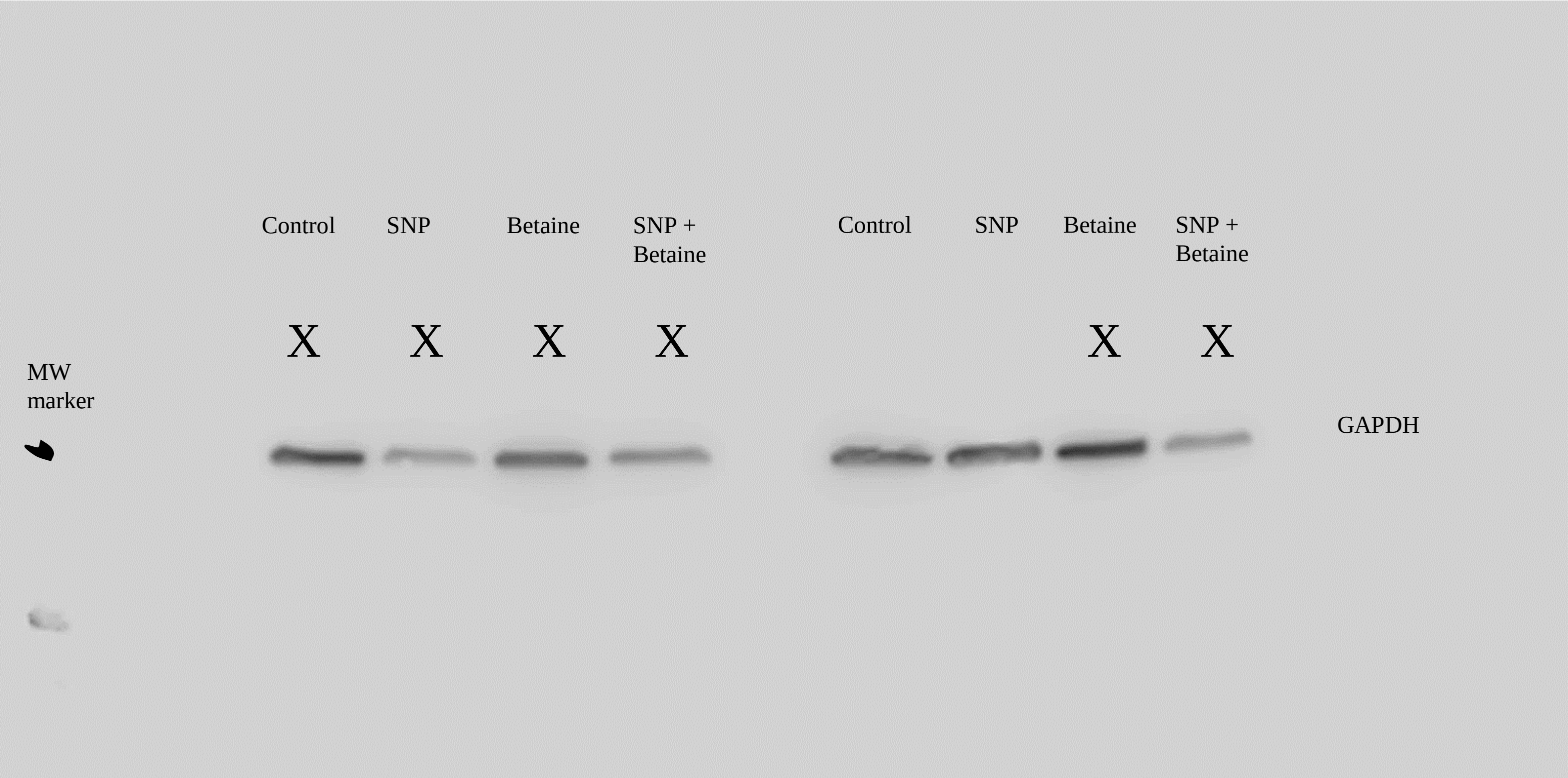

MO3.13 cells treated with 400 µM SNP and/or 1 mM betaine. Whole cell lysate made with 1x RIPA. Samples run on 10% gel in duplicate. Image captured on ImageQuant LAS 400. Figure 3A.

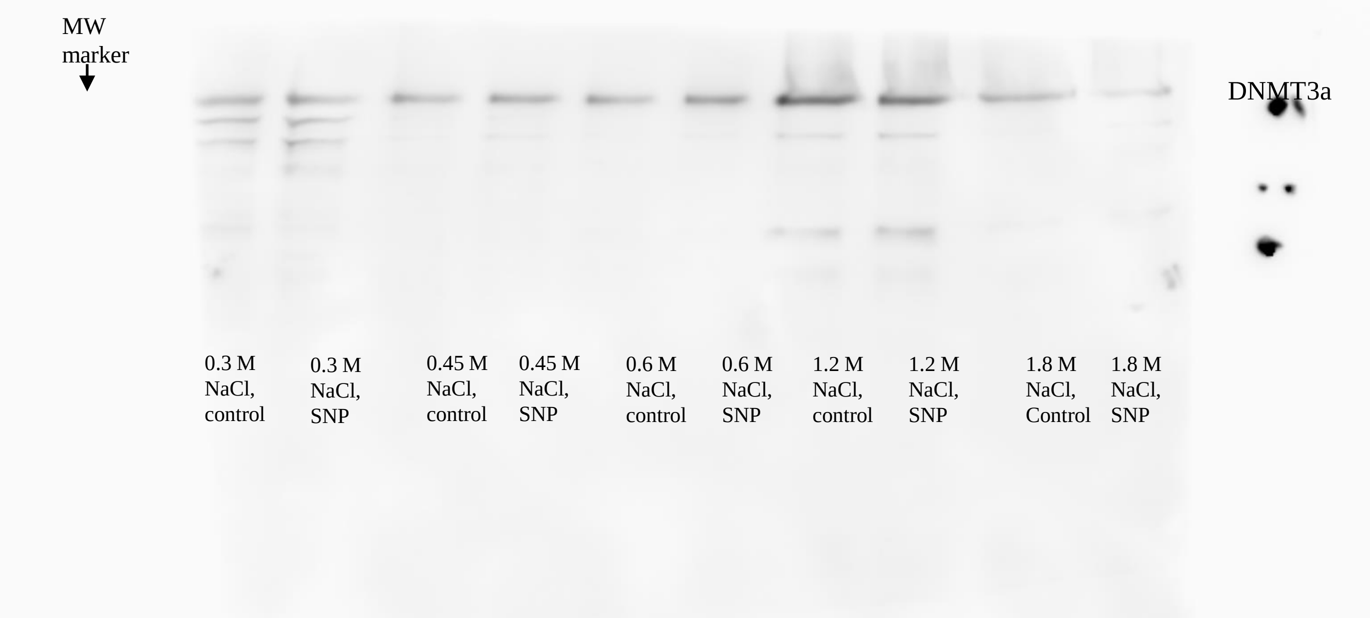

MO3.13 cells, control and treated with 400 uM SNP, salt fractionated. Samples run on 12% gel. Image captured on ImageQuant LAS 400. Figure 4A.

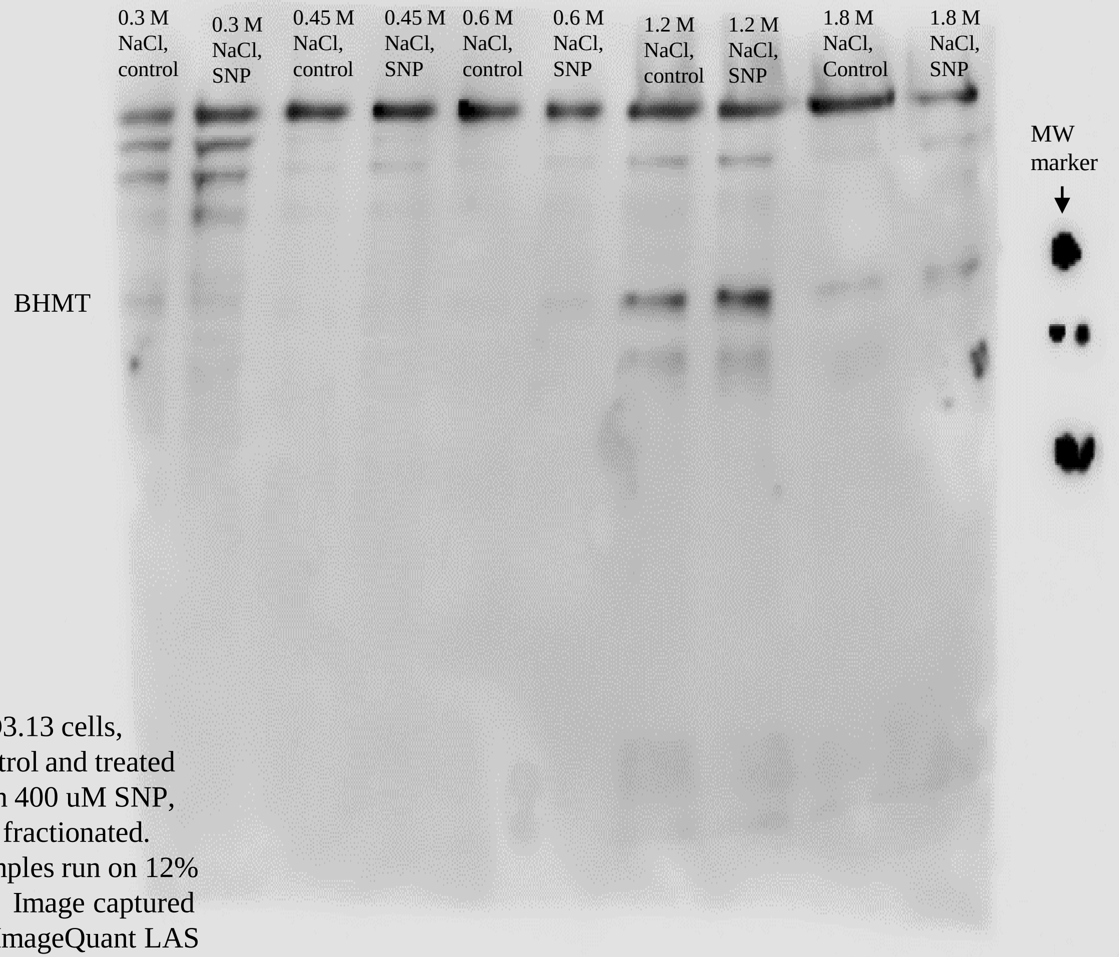

MO3.13 cells, control and treated with 400 uM SNP, salt fractionated. Samples run on 12% gel. Image captured on ImageQuant LAS 400. Figure 4A.

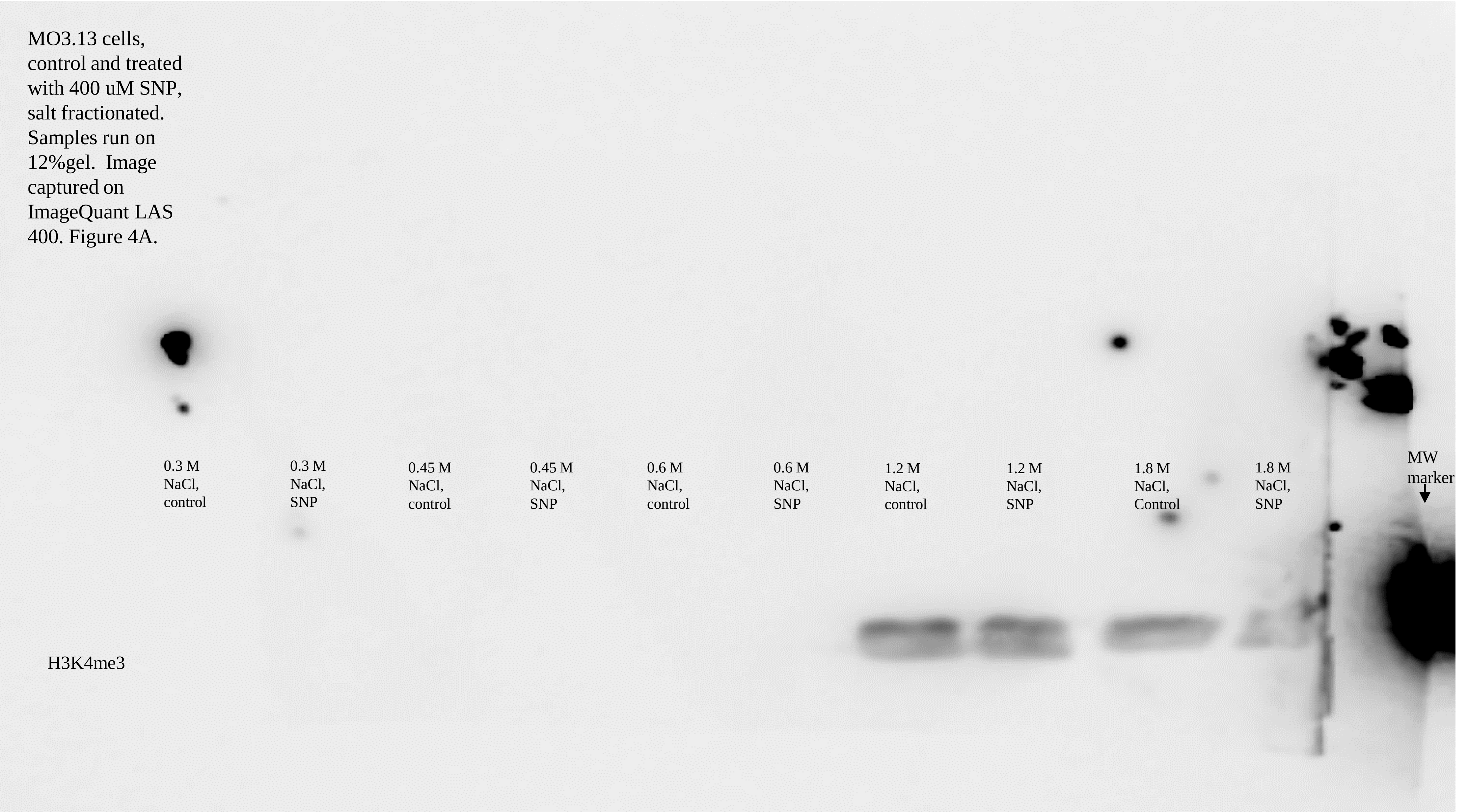
MO3.13 cells, control and treated with 400 uM SNP, salt fractionated. Samples run on 12%gel. Image captured on ImageQuant LAS 400. Figure 4A.
0.3 M NaCl, control
0.3 M NaCl, SNP
0.45 M NaCl, control
0.45 M NaCl, SNP
0.6 M NaCl, control
0.6 M NaCl, SNP
1.2 M NaCl, control
1.2 M NaCl, SNP
1.8 M NaCl, Control
1.8 M NaCl, SNP
MW marker
H3K4me3

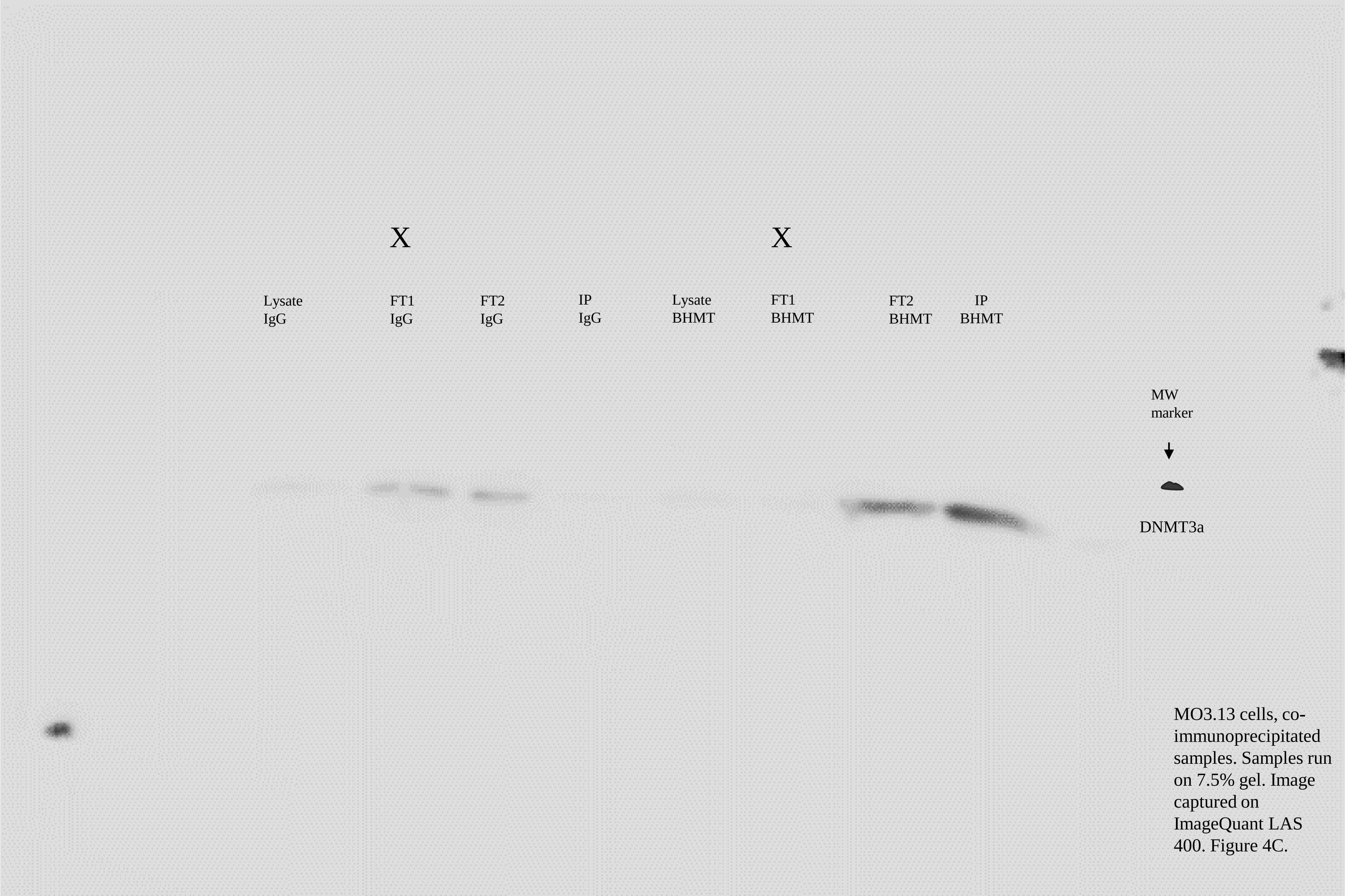

MO3.13 cells, co-immunoprecipitated samples. Samples run on 7.5% gel. Image captured on ImageQuant LAS 400. Figure 4C.

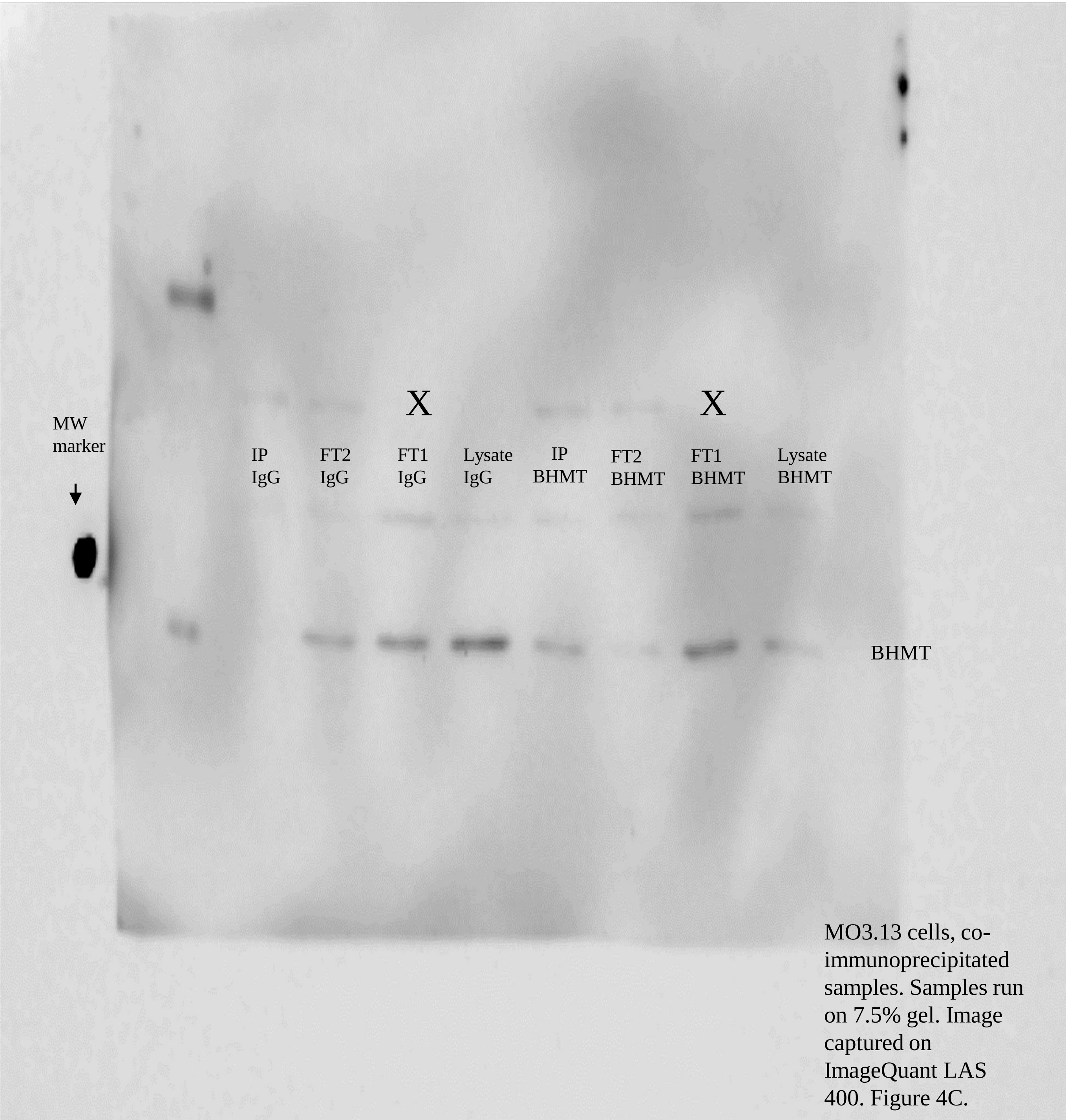

MO3.13 cells, co-immunoprecipitated samples. Samples run on 7.5% gel. Image captured on ImageQuant LAS 400. Figure 4C.

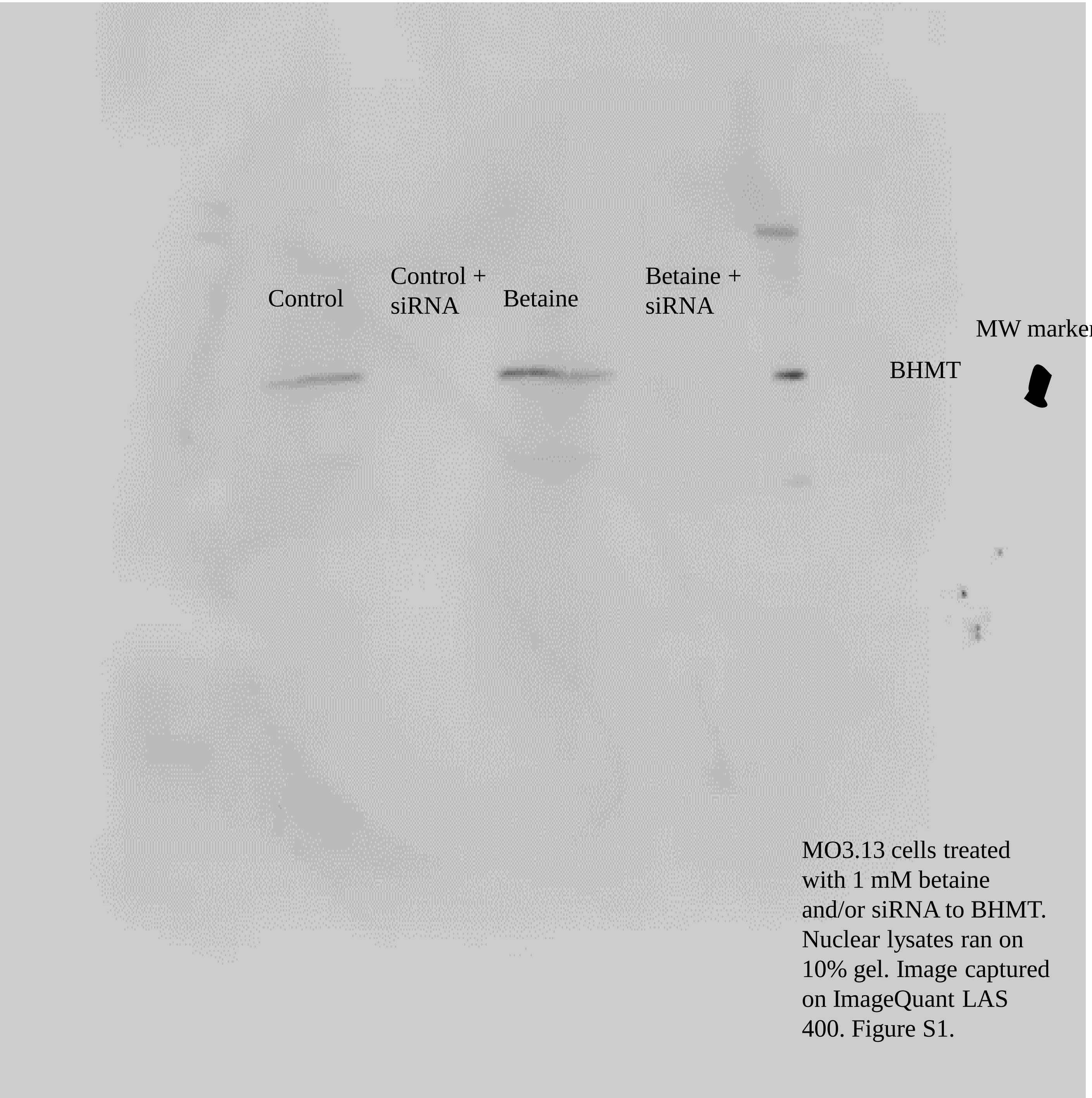

MO3.13 cells treated with 1 mM betaine and/or siRNA to BHMT. Nuclear lysates ran on 10% gel. Image captured on ImageQuant LAS 400. Figure S1.

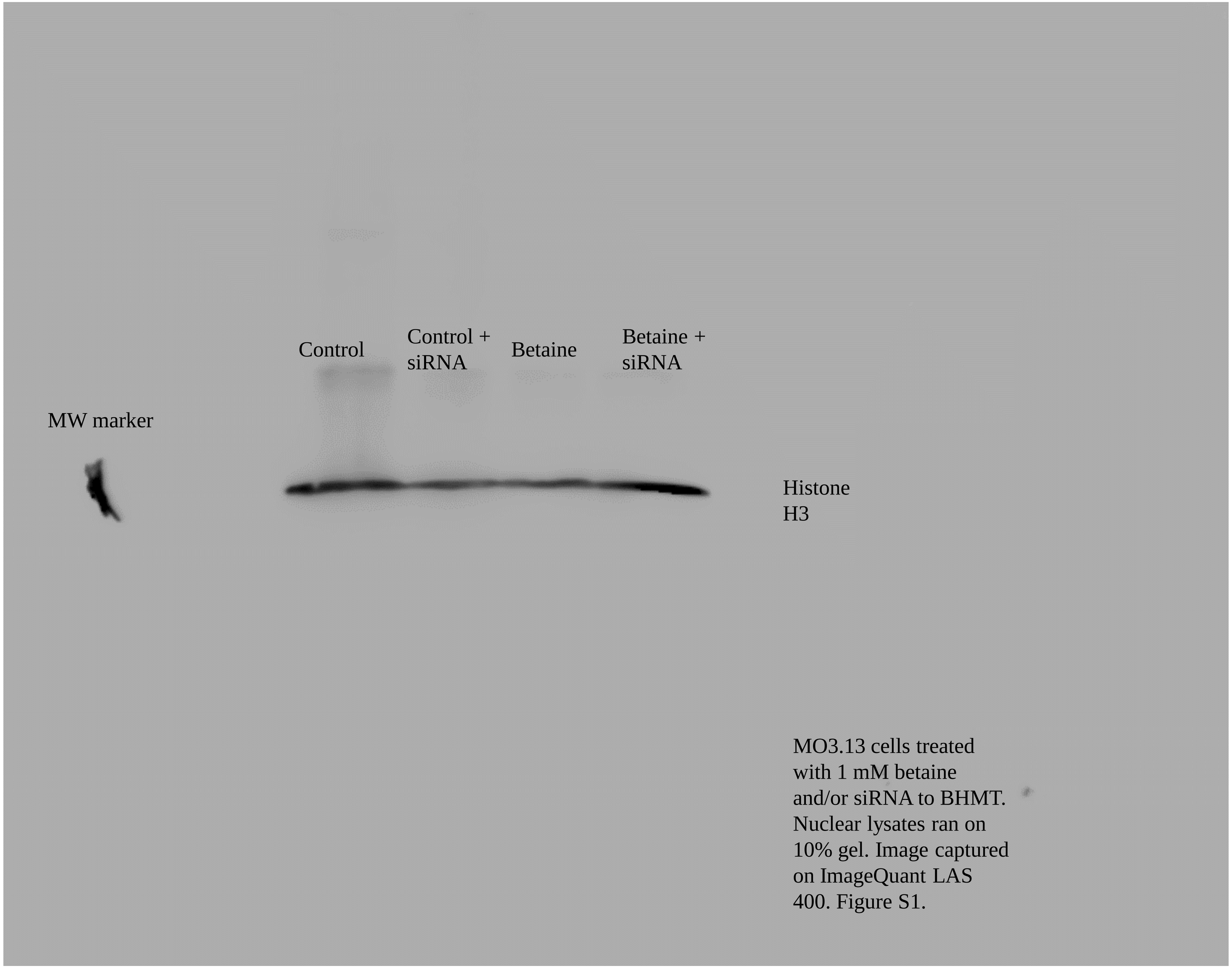

MO3.13 cells treated with 1 mM betaine and/or siRNA to BHMT. Nuclear lysates ran on 10% gel. Image captured on ImageQuant LAS 400. Figure S1.
